# Therapeutic Potential of Lymphatic Endothelial Progenitor Cells in Secondary Lymphedema: A Preclinical Murine Study

**DOI:** 10.3390/biomedicines14081782

**Published:** 2026-08-07

**Authors:** Ibon Jaunarena, María-Teresa Iglesias-Gaspar, Inazio Arriola-Alvarez, Ander Izeta, Irene Diez-Itza, Arantza Lekuona, Héctor Lafuente

**Affiliations:** 1 Obstetrics and Gynecology Group, Biogipuzkoa Health Research Institute, 20014 San Sebastián, Spain; 2 Osakidetza Basque Health Service, Donostia University Hospital, 20014 San Sebastián, Spain; 3 Department of Medical-Surgical Specialties, University of the Basque Country (UPV/EHU), 48940 Leioa, Spain; 4 Faculty of Health Sciences, University of Deusto, 20018 San Sebastián, Spain; 5 Stem Cells & Aging Group, Biogipuzkoa Health Research Institute, 20014 San Sebastián, Spain; 6 School of Engineering, Tecnun, University of Navarra, 20009 San Sebastián, Spain

**Keywords:** secondary lymphedema, regenerative medicine, lymphatic endothelial progenitor cells, cell therapy, lymphatic regeneration, mouse tail lymphedema model, lymphangiogenesis

## Abstract

**Background**: Secondary lymphedema is a chronic complication of oncologic surgery and radiotherapy for which effective disease-modifying therapies remain lacking. While mesenchymal stem cells (MSCs) have shown partial benefit in experimental models, direct functional comparison with lineage-committed lymphatic endothelial progenitor cells (LEPCs) remains limited. **Methods**: Secondary lymphedema was induced in C57BL/6J mice by circumferential tail skin excision with disruption of superficial lymphatics. Animals received intradermal phosphate-buffered saline (PBS), MSCs, or adipose-derived LEPCs on postoperative days 1 and 7. Lymphatic function was longitudinally quantified using IVIS-based near-infrared indocyanine green (ICG) imaging with standardized region-of-interest analysis. Tail diameter was measured serially throughout follow-up as a complementary morphometric parameter. Tissue remodeling was assessed by Picrosirius Red staining and qualitative LYVE-1 immunofluorescence. Statistical analysis incorporated mixed-effects modeling to evaluate treatment group, sex, time, and their interaction terms, with estimation of effect sizes and 95% confidence intervals. **Results**: LEPC-treated mice demonstrated higher IVIS signal intensity than MSC- and PBS-treated animals, consistent with improved lymphatic transport at the injury site. Longitudinal tail diameter analysis showed a more favorable temporal profile in the LEPC group relative to controls, with exploratory analysis suggesting potential variations in temporal profiles between sexes that warrant further investigation in larger cohorts. Histologic analysis showed reduced non-tissue/void area and a more favorable qualitative pattern of LYVE-1 staining in LEPC-treated tissue. **Conclusions**: LEPC administration enhanced functional and structural recovery in a murine model of secondary lymphedema. These findings support further translational evaluation of LEPC-based approaches as a regenerative strategy for secondary lymphedema.

## 1. Background

Secondary lymphedema is a chronic consequence of lymphatic system failure, in which sustained impairment of fluid transport drives progressive swelling, tissue fibrosis, and loss of function. Among cancer survivors, particularly those treated for gynecologic and breast malignancies, it represents a frequent and long-lasting complication that substantially affects quality of life [1,2,3,4].

The pathophysiology involves persistent lymphatic injury combined with chronic inflammation and maladaptive tissue remodeling. Accumulation of interstitial fluid is associated with an IL-4/IL-13–driven fibrotic and adipogenic response that compromises lymphatic vessel repair and valve integrity [5]. Within this environment, LEPCs participate in vascular regeneration, although their reparative capacity may be limited by the profibrotic milieu. Recent evidence further suggests that mesenchymal progenitors can acquire a lymphatic endothelial phenotype through PROX1-dependent pathways without requiring a venous intermediate stage [6], highlighting developmental plasticity within lymphangiogenic precursor populations. This plastic and specialized nature of lymphatic endothelium is further exemplified by recent discoveries of functional lymphatic vessels within bone tissue and the skeletal microenvironment, which play critical roles in tissue repair, immune regulation, and homeostasis [7].

Current management strategies remain largely symptomatic rather than curative. Complex decongestive therapy, including manual lymphatic drainage, compression therapy, exercise, and skin care, continues to be the cornerstone of care despite requiring lifelong adherence and offering variable clinical benefit [4,8]. Microsurgical procedures such as lymphovenous anastomosis (LVA) and vascularized lymph node transfer (VLNT) may reduce limb volume and improve function, but they are technically demanding and do not consistently restore physiologic lymphatic flow [9,10,11,12]. These limitations underscore the need for therapies that directly target the biological mechanisms of lymphatic regeneration.

Regenerative strategies have therefore gained attention as potential means to reconstruct damaged lymphatic networks. Preclinical and early clinical studies indicate that cell-based approaches may promote lymphangiogenesis, modulate inflammation, and attenuate fibrosis, thereby improving lymphatic drainage [13,14]. MSCs and adipose-derived stem cells (ADSCs) have been the most extensively investigated, primarily through paracrine support of host tissue repair [15]. Early-phase clinical studies have demonstrated the feasibility and safety of ADSC-based interventions for cancer-related lymphedema [16,17]. However, clinical outcomes remain variable, suggesting that more lineage-oriented or mechanism-informed approaches may be required.

Recent experimental advances have expanded the therapeutic landscape of adipose-derived cells. Modulation of the Hippo–YAP pathway in ADSCs has been shown to enhance lymphangiogenesis and reduce fibrosis in murine tail lymphedema models [18]. In parallel, LEPCs have emerged as promising candidates for lymphatic regeneration because of their capacity to differentiate toward mature lymphatic endothelial phenotypes and support new vessel formation [5,6,19,20]. While uncommitted MSCs act broadly via paracrine anti-inflammatory signaling, LEPCs possess an innate lineage-specific commitment that theoretically bypasses the need for local differentiation cues, providing a more direct and efficient cellular building block for neo-lymphangiogenesis.

Despite these encouraging findings, rigorous comparative evaluation of LEPC-based therapy in clinically relevant models of secondary lymphedema remains limited. In particular, their functional performance relative to MSCs in validated preclinical systems has not been fully defined. This gap highlights the importance of carefully designed studies integrating quantitative functional imaging with structural and histologic endpoints.

Accordingly, the present study investigated the therapeutic effect of adipose-derived LEPCs in a validated mouse model of secondary lymphedema. By integrating longitudinal IVIS–ICG functional imaging, serial morphometric assessment, and histological quantification of tissue void spaces and LYVE-1+ vessel structures, we provide a comprehensive preclinical evaluation of LEPC-associated tissue recovery. These findings aim to inform future translational development of lineage-oriented cell strategies for secondary lymphedema [15,16,18].

## 2. Materials and Methods

### 2.1. Study Design

This preclinical experimental study was designed to evaluate the therapeutic potential of adipose-derived LEPCs for secondary lymphedema. We employed a validated mouse tail model that induces sustained lymphatic drainage impairment and reproduces the main pathological features of human lymphedema, including fluid stasis, inflammatory activation, and progressive fibrotic remodeling [21,22,23].

To generate this condition, standardized surgical injury consisting of full-thickness circumferential skin excision combined with disruption of the superficial lymphatic collectors was performed, following established methodologies for inducing stable lymphatic dysfunction in vivo [21,22,23]. Treatments were administered on postoperative days 1 and 7 according to randomized treatment assignment. Because several animals met predefined welfare or data-quality exclusion criteria during follow-up, the final analysed cohort was smaller than the initially allocated population.

The therapeutic response was evaluated through a multimodal approach combining near-infrared indocyanine green (ICG) imaging via the IVIS SpectrumCT system, serial measurements of tail diameter, and detailed histological assessment of lymphatic marker expression and extracellular matrix remodeling. This integrative design enabled a comprehensive characterization of functional and structural regeneration following LEPC treatment [23,24].

### 2.2. Animal Model

A total of 24 C57BL/6J mice were allocated to one of the three experimental treatment groups and underwent longitudinal follow-up. These comprised male and female C57BL/6J mice, 8 weeks old and weighing 20–25 g, obtained from Janvier Labs (Le Genest-Saint-Isle, France). The animals were acclimatized for at least 7 days before experimentation and housed under specific pathogen-free (SPF) conditions in groups of 3–5 per cage. The environment was controlled for temperature (22 ± 2 °C), humidity (45–65%), and a 12:12 h light/dark cycle, with ad libitum access to food and water. All the cages were provided with nesting material and shelters to increase their welfare.

All procedures complied with Spanish Royal Decree 53/2013 and the European Directive 2010/63/EU. The study protocol was approved by the Ethics Committee for Animal Experimentation of IIS BioGipuzkoa and the competent authority of Gipuzkoa (OH-23-25). The animals were checked daily during the first postoperative week and at least three times weekly thereafter. Humane endpoints included >15% body weight loss, persistent infection or necrosis, impaired mobility, or signs of sustained distress. Euthanasia was performed under deep isoflurane anesthesia followed by cervical dislocation, following established welfare recommendations [25]. The study design, conduct, and reporting followed the ARRIVE 2.0 recommendations for in vivo research [26].

### 2.3. Mouse Tail Lymphedema Model

Secondary lymphedema was induced via a standardized and stereomicroscope-assisted surgical protocol. A narrow full-thickness circumferential strip of dorsal tail skin (approximately 2 mm wide) was carefully removed 1–2 cm distal to the tail base to interrupt the superficial lymphatic collectors. To facilitate the identification of lymphatic vessels, 25 µL of 1% Evans blue dye (Sigma-Aldrich, St. Louis, MO, USA) was injected intradermally into the distal tail, allowing selective visualization and subsequent ligation of marked lymphatic channels via fine Vannas microscissors. This technique allowed reproducible interruption of superficial lymphatics while preserving major blood vessels, thereby minimizing ischemic artifacts.

Anesthesia was induced with 4–5% isoflurane and maintained at 1–2% in oxygen (0.5–1 L/min). Perioperative analgesia was provided with subcutaneous meloxicam (0.2 mg/kg). No postoperative bandaging was applied to avoid irritation or self-mutilation behaviors. Lymphedema development was confirmed by a persistent increase in tail diameter measured 1 cm distal to the excision site, following established criteria.

This protocol induces sustained impairment of lymphatic drainage and is associated with progressive interstitial fluid accumulation, inflammatory activation, fibroadipose remodeling, and delayed endogenous lymphangiogenesis, thereby recapitulating key pathological features of human secondary lymphedema [21,22,23]. Recent ultrastructural studies further confirmed that this model reproduces the intussusceptive and sprouting lymphangiogenesis patterns observed in human disease and in high-resolution murine regeneration models [27]. For these reasons, the mouse tail lymphedema model remains a widely used and technically robust platform for evaluating lymphatic dysfunction and testing regenerative therapies in vivo.

### 2.4. Cell Isolation and Characterization

#### 2.4.1. Mesenchymal Stem Cells (MSCs)

All MSCs used in this study were murine adipose-derived cells. No human-derived cells or human material were used.

Adipose-derived MSCs were obtained from the stromal vascular fraction of murine adipose tissue following enzymatic and mechanical dissociation. Briefly, excised adipose tissue was finely minced and subjected to collagenase type XI digestion (1 mg/mL, 60 min, 37 °C) with gentle agitation to release stromal cells. The digested material was filtered through a 70-µm cell strainer to remove debris and centrifuged to isolate the stromal vascular pellet [28].

The resulting cell fraction was resuspended and expanded directly onto the plastic plates with complete Mesencult medium (05513, StemCell, Vancouver, BC, Canada) and cultured in a humidified incubator with 5% CO_2_ at 37 °C. Cultures were maintained until they reached approximately 70–80% confluence and were used at passage 3. Cell viability (>90%) was confirmed prior to administration.

#### 2.4.2. Lymphatic Endothelial Progenitor Cells (LEPCs)

At passage 3, MSCs were detached using 0.25% Trypsin-EDTA, seeded at 20,000 cells/cm^2^ in complete mouse endothelial cell medium (ECGM, M1168, Cell Biologics, Chicago, IL, USA) supplemented with 50 ng/mL of VEGF-C (752-VC, R&D Systems, Minneapolis, MN, USA), and cultured for 7 days under standard conditions (37 °C, 5% CO_2_), following a selection strategy previously detailed and fully characterized for lymphatic lineage markers (such as PROX1, VEGFR3, PDPN, and LYVE1) in adipose-derived sub-populations by our research group [29]. While the isolation and lineage-selection protocols were implemented following our established and validated laboratory methodology, batch-specific post-sorting purity percentages, quantitative surface marker gating profiles, and batch-to-batch variability data were not recorded for these specific experimental cell lots.

Subsequently, podoplanin-positive (Pod^+^) cells were sorted, briefly expanded (≤passage 3), and characterized by immunofluorescence and flow cytometry for canonical lymphatic markers, including podoplanin and LYVE-1. Only cell preparations with viability >90% were used for in vivo administration.

### 2.5. Experimental Groups, Randomization, and Blinding

Animals allocated to the efficacy experiment were randomized to three treatment groups using a sex-stratified sealed-envelope method: PBS control, MSC, or LEPC. Initial allocation included 8 animals per group, with an equal sex distribution within each group (4 females and 4 males). The allocation sequence was concealed from investigators involved in follow-up assessments by using sealed opaque envelopes. During follow-up, animals were excluded from the final longitudinal analysis if they met humane endpoints or if complete serial tail-diameter measurements could not be obtained because of tail necrosis, tail loss, or infection. The final analysed cohort consisted of 17 animals: PBS control group (*n* = 4), MSC group (*n* = 7), and LEPC group (*n* = 6). Treatments were administered on postoperative days 1 and 7. A total volume of 100 µL (1 × 10^6^ cells in suspension or PBS) was delivered through five intradermal microinjections evenly distributed around the excision site via a 30-gauge needle to ensure uniform coverage of the lymphatic injury zone.

Randomization was performed by an investigator not involved in the follow-up assessments. Tail measurements, IVIS/ICG imaging, and histological analysis were conducted by blinded examiners in accordance with best practices for minimizing experimental bias in animal research [26,30].

### 2.6. Functional Assessment

#### Tail Diameter Measurements

Tail diameter was recorded before treatment (baseline) and serially throughout the 36-day follow-up using a digital micrometer. Measurements were consistently obtained 1 cm distal to the surgical site to ensure reproducibility. This approach represents a quantitative and widely implemented method for monitoring lymphedema progression and resolution in validated murine tail models [21,22,23].

### 2.7. Lymphatic Function Assessment

#### Indocyanine Green (ICG) Near-Infrared Fluorescence Imaging (IVIS)

Lymphatic transport was assessed using near-infrared ICG imaging. Mice received 25 µL of ICG (1 mg/mL in PBS; Sigma-Aldrich) intradermally at the tail tip and were imaged at the predefined post-treatment time points using the IVIS SpectrumCT system (PerkinElmer, Shelton, CT, USA). Fluorescence was acquired using 745/800 nm excitation/emission settings. Quantification was performed in Living Image software version 4.8.4 (PerkinElmer, Waltham, MA, USA) using a standardized circular region of interest (ROI) placed at the level of the surgical interruption based on consistent anatomical landmarks across animals. Total radiant efficiency within the ROI was recorded as the quantitative readout of tracer signal. This approach enabled standardized longitudinal assessment of lymphatic transport in the murine tail model [24]. Negative values after background subtraction were interpreted as absence of detectable signal and set to 0 for descriptive group summaries.

### 2.8. Histological Analysis

On day 36, the mice were euthanized, and tail tissues encompassing the injury site were harvested for structural analysis. The samples were fixed in 4% paraformaldehyde (24 h at 4 °C), decalcified, dehydrated, embedded in paraffin, and sectioned at 5 µm. The samples were stained with hematoxylin and eosin to assess overall tissue architecture and cellular morphology.

#### 2.8.1. Interstitial Tissue Architecture

Collagen deposition was assessed by Picrosirius Red staining (Sigma-Aldrich) and visualized under polarized light. Picrosirius Red-stained sections were utilized to segment and quantify the percentage of non-tissue/void area within the ROI, serving as a histological surrogate for structural tissue disruption and interstitial fluid accumulation rather than direct collagen densitometry, and was performed using ImageJ software version 2.16.0 (NIH, Bethesda, MD, USA) with standardized color threshold algorithms. It is a consistent pathological feature in murine tail lymphedema models and has been quantified in prior studies using comparable histological approaches [22].

#### 2.8.2. Lymphatic Marker Assessment

Immunofluorescence staining was performed using antibodies against LYVE-1 (Abcam, Cambridge, UK), followed by Alexa Fluor-labeled secondary antibodies and DAPI counterstaining. Images were acquired with a Leica SP8 confocal microscope (Wetzlar, Germany). LYVE-1 staining was assessed qualitatively for the presence of lymphatic structures and vessel-like organization within the excision region. Histologic evaluation of lymphatic structures has been previously described in the murine tail lymphedema model [31]. Due to technical constraints associated with notable background fluorescence noise, automated quantification of LYVE-1 signal intensity or vessel counts could not be reliably achieved. Consequently, LYVE-1 staining was evaluated qualitatively by a blinded investigator to assess general structural patterns.

### 2.9. Statistical Analysis

Statistical analyses were conducted using STATA SE v.18.0 (StataCorp, College Station, TX, USA). Descriptive data are presented as mean ± standard deviation (SD). As an initial exploratory step, tail diameter was summarized by treatment group, sex, and time point. Individual longitudinal trajectories were plotted to visualize within-animal variability over time, and model-based predicted trajectories were generated stratified by treatment group and sex.

The primary longitudinal endpoint, tail diameter, was analysed using a linear mixed-effects model to account for the repeated-measures structure of the data. The model included treatment group, sex, time, and their interaction terms as fixed effects, including group × sex, group × time, sex × time, and group × sex × time interactions. A random slope for time was specified at the individual animal level to account for between-animal heterogeneity in temporal evolution. A random intercept was not retained after model assessment indicated that its inclusion was not required. This modelling strategy was selected because it accounts for repeated measurements within animals and is suitable for unbalanced longitudinal datasets resulting from unequal group sizes after follow-up exclusions.

Model-based predictions with 95% confidence intervals were generated to illustrate temporal patterns across treatment groups and sex strata. Normality was assessed using the Shapiro–Wilk test [32]. For comparisons of single time-point endpoints, unpaired Student’s *t* tests were applied to normally distributed data, and the Mann–Whitney U test was used for nonparametric distributions. Comparisons across three groups were performed with one-way ANOVA followed by Tukey’s post hoc test [33] or, when appropriate, with the Kruskal–Wallis test and Dunn’s multiple comparisons [34]. Outliers were assessed using Grubbs’ test [35]. Key outcomes were additionally reported with effect sizes and 95% confidence intervals. Sample size selection was guided by prior studies using the murine tail lymphedema model [21,23] and aligned with recommendations for rigorous preclinical research [26,30]. Statistical significance was set at *p* < 0.05.

## 3. Results

Out of the 24 initially randomized mice (*n* = 8 per group), 7 animals were excluded due to meeting humane endpoints or technical complications: PBS control (*n* = 4 excluded due to tail necrosis between days 4 and 11), MSC group (*n* = 1 excluded due to localized infection on day 7), and LEPC group (*n* = 2 excluded due to tail loss/necrosis by day 14). This resulted in a final analyzed cohort of 17 mice (PBS: *n* = 4; MSC: *n* = 7; LEPC: *n* = 6).

### 3.1. Tail Swelling over Time (Tail Diameter)

Tail diameter was measured serially throughout the 36-day follow-up after lymphedema induction. Descriptive tail-diameter values stratified by treatment group, sex, and time point are shown in Table 1.

**Figure 1:**
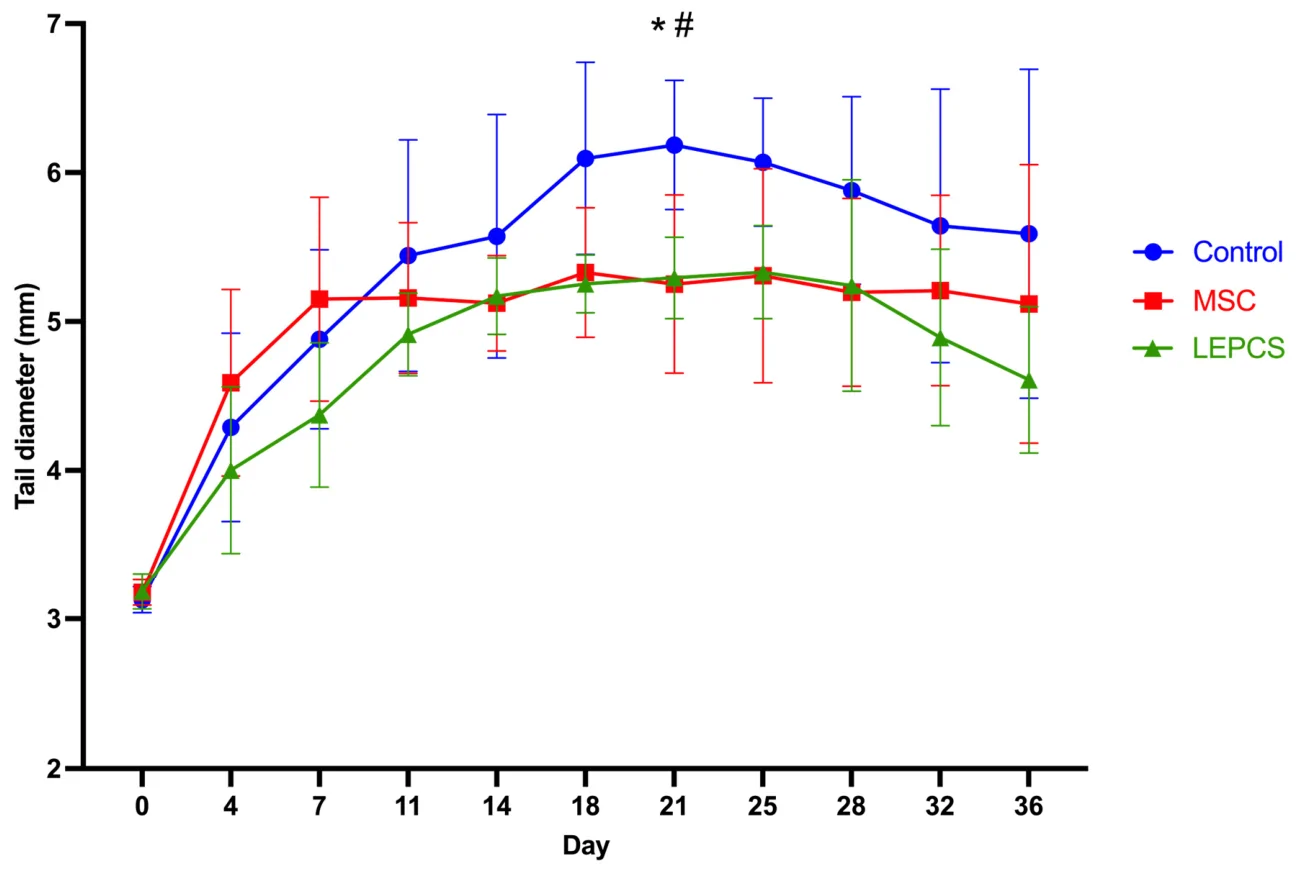
Longitudinal progression of observed tail diameter (Mean ± SD) across treatment groups. Tail diameter trajectories were modelled using a linear mixed-effects model including treatment group, sex, day, and interaction terms. Curves represent model-based predicted values with 95% confidence intervals. (* vs. PBS control, # vs. MSC; evaluated via post-hoc comparisons, significance at *p* < 0.05).

All animals developed increased tail diameter after surgery, with visible differences in the magnitude and temporal evolution of swelling across treatment groups (Figure 1). Baseline diameters were comparable among groups: control (3.13 ± 0.08 mm), MSC (3.18 ± 0.10 mm), and LEPC (3.19 ± 0.11 mm). Peak swelling was greatest in the control group (6.09 ± 0.55 mm), whereas MSC-treated animals reached a lower maximum diameter (5.33 ± 0.36 mm) and LEPC-treated mice showed the mildest peak swelling (5.25 ± 0.23 mm). By day 36, the LEPC group also exhibited the lowest final tail diameter (4.61 ± 0.47 mm), compared with the MSC (5.12 ± 0.59 mm) and control (5.59 ± 0.55 mm) groups.

**Figure 2:**
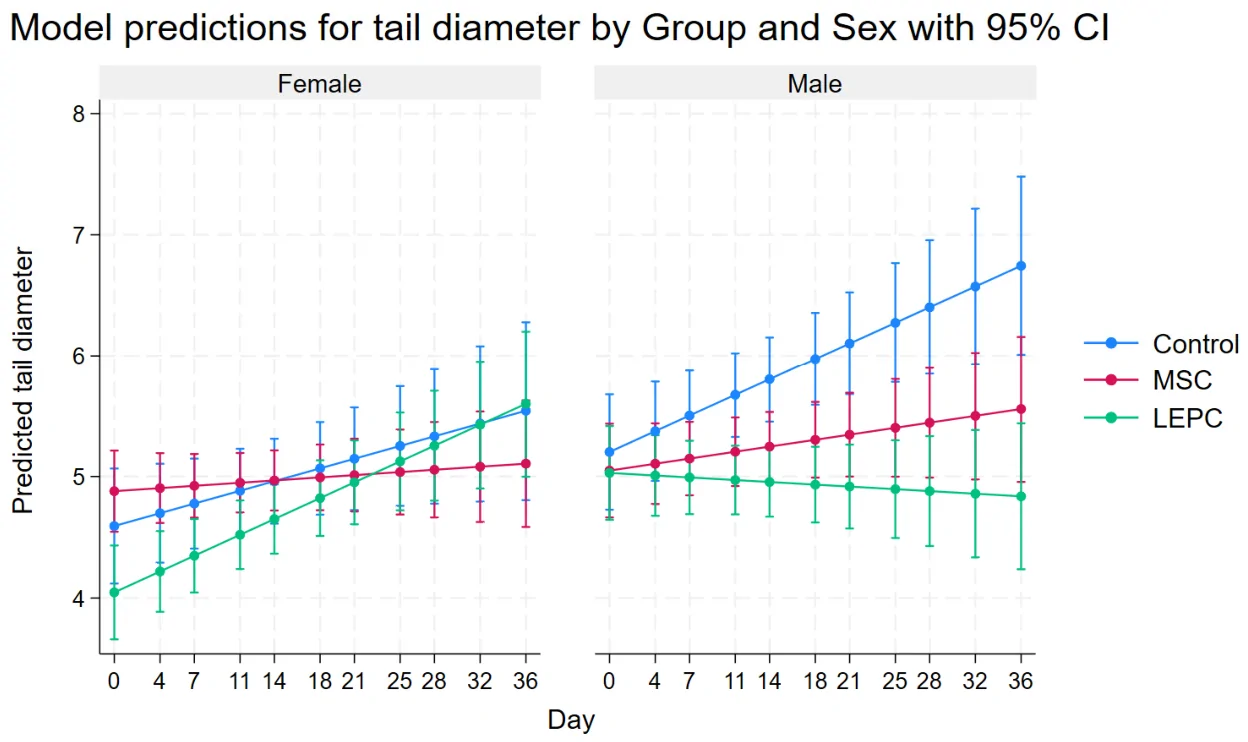
Model-based predicted tail diameter trajectories generated from the parsimonious linear mixed-effects model adjusting for sex as a covariate. Predicted tail diameter values were generated from the linear mixed-effects model including treatment group, sex, day, and their interaction terms. Curves show the estimated temporal evolution of tail diameter across treatment groups separately for male and female mice, with 95% confidence intervals. The model suggests a flatter temporal trajectory in LEPC-treated males compared with control males, whereas this pattern was less evident in females.

Longitudinal analysis using a linear mixed-effects model including treatment group, sex, time, and their interaction terms showed a differential temporal evolution of tail diameter across treatment groups and sex strata (Table 2 and Figure 2). In the reference stratum, corresponding to LEPC-treated males, the temporal slope was small and not statistically significant, indicating an almost flat trajectory over follow-up. Compared with LEPC-treated males, control males showed a significantly steeper increase in tail diameter over time, whereas MSC-treated males showed an intermediate, non-significant trend. A significant sex × time interaction was also observed within the LEPC group, indicating a steeper temporal increase in LEPC-treated females than in LEPC-treated males. Significant group × sex × time interactions further suggested that sex modified the effect of treatment group on tail-diameter evolution.

As a complementary descriptive analysis, the difference between each animal’s maximum tail diameter and baseline (ΔMax–Day 0) was also examined. The control group showed the greatest increase (3.33 ± 0.46 mm), followed by the MSC (2.68 ± 0.47 mm) and LEPC (2.45 ± 0.51 mm) groups. Although the difference between the control and LEPC groups did not reach statistical significance in the nonparametric comparison (*p* = 0.094), the descriptive pattern was consistent with the overall temporal trend observed in LEPC-treated animals.

### 3.2. Histological Assessment of Tissue Remodeling (Fibrosis and Non-Tissue/Void Spaces)

Interstitial tissue composition was quantified using the WSI Sirius Red Manual workflow. Within each region of interest (ROI), Sirius Red-positive area and non-tissue/void area were segmented and expressed as percentages of the total ROI.

**Figure 3:**
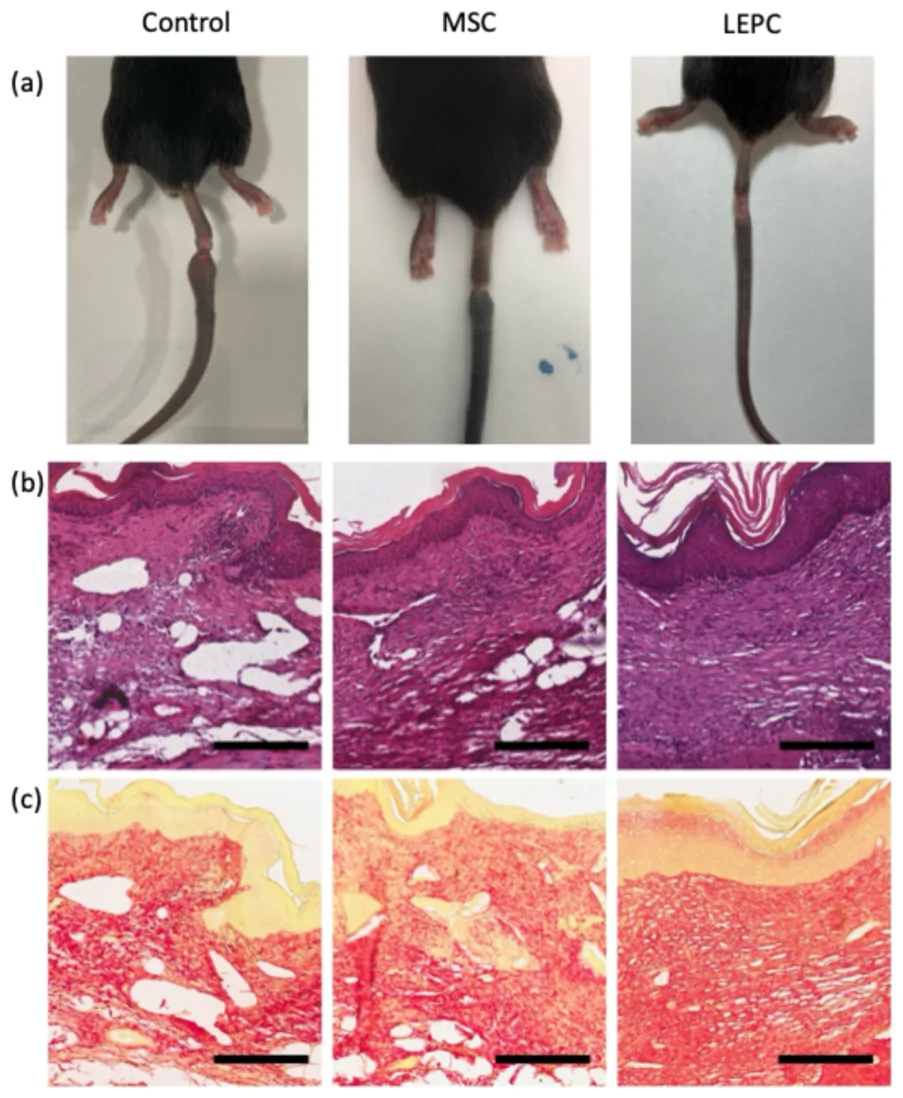
Histological assessment of tissue remodeling. (**a**) Representative macroscopic view of the mouse tail on day 36 after lymphedema induction. (**b**) Hematoxylin and eosin-stained section of the injury area showing overall tissue architecture. (**c**) Picrosirius Red-stained section used to assess collagen deposition and quantify non-tissue/void spaces within the region of interest. Scale bar, 200 µm.

Quantitative analysis showed a significantly lower non-tissue/void area in LEPC-treated mice (7.37 ± 2.87%) than in MSC-treated (15.87 ± 2.64%) and control animals (19.62 ± 9.31%). No significant difference was observed between the MSC and control groups (*p* = 0.548). In contrast, the non-tissue/void area was significantly reduced in the LEPC group compared with both the control group (*p* = 0.048) and the MSC group (*p* = 0.002). Representative histological images are shown in Figure 3. These findings indicate a more preserved tissue architecture in LEPC-treated animals and are consistent with the reduced tail swelling observed during longitudinal follow-up.

### 3.3. Lymphatic Function Assessment

Near-infrared ICG imaging was quantified using a standardized circular ROI placed at the level of the surgical interruption. Quantitative analysis of total radiant efficiency showed higher IVIS signal intensity in LEPC-treated animals (5.26 × 10^9^ ± 2.46 × 10^9^) than in MSC-treated mice (5.97 × 10^8^ ± 1.00 × 10^9^) and PBS-treated controls (7.88 × 10^7^ ± 9.79 × 10^7^). Overall differences among groups were significant (Kruskal–Wallis, *p* = 0.0046). Pairwise comparisons showed higher signal intensity in LEPC-treated animals than in MSC-treated mice (*p* = 0.0051) and PBS-treated controls (*p* = 0.0139), whereas no significant difference was observed between MSC-treated and PBS-treated animals (*p* = 0.6202). Low-level signal detected in some PBS- and MSC-treated animals was attributable to background ROI capture rather than biologically meaningful tracer transport.

### 3.4. Lymphatic Vessel Regeneration

**Figure 4:**
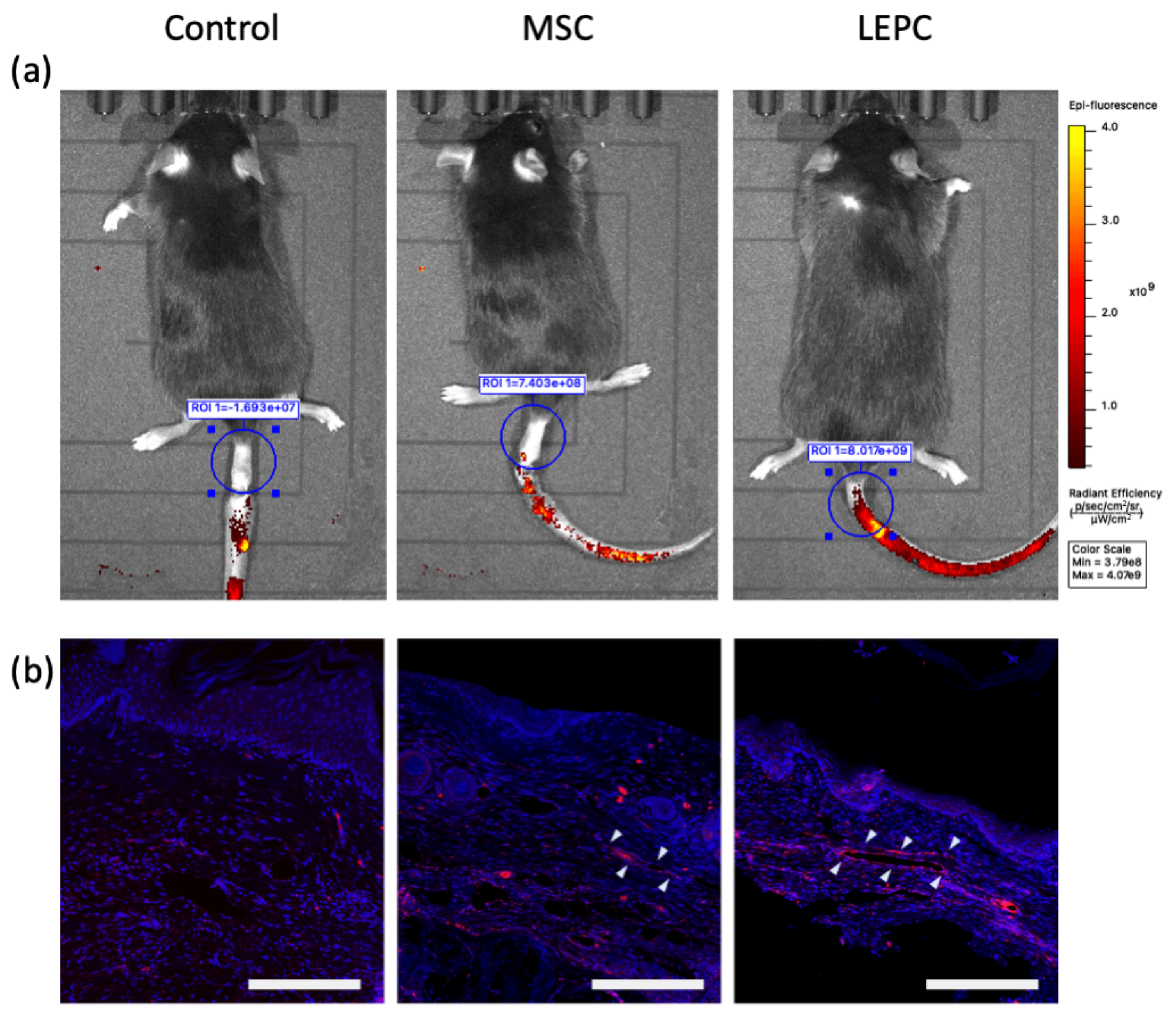
Evaluation of functional lymphatic drainage (IVIS–ICG) and structural tissue patterns (LYVE-1 immunofluorescence). (**a**) Representative IVIS–ICG near-infrared fluorescence images on day 36 after lymphedema induction in PBS-(*n* = 4), MSC-(*n* = 7), and LEPC-treated (*n* = 6) animals. Functional ICG signal retention was captured 30 min post-intradermal injection. Quantification was performed using total radiant efficiency measured within a standardized circular ROI of 1 cm in diameter placed at the level of the surgical interruption. Fluorescence intensity scale bars represent Radiant Efficiency [p/s/cm^2^/sr]/[μW/cm^2^]. (**b**) Representative confocal images of LYVE-1 immunostaining (Alexa Fluor 647/ far-red channel; DAPI nuclear counterstain in blue) in the excision region on day 36. Analysis was performed on 3 cross-sections per animal (sampled at 5 mm, 10 mm, and 15 mm distal to the surgical transection site; 3 representative high-power fields/ROIs per section). Arrows indicate LYVE-1-positive tissue elements. Scale bar: 200 µm. Note on histological evaluation: Due to inherent background tissue autofluorescence in the murine tail model, LYVE-1 immunostaining was evaluated qualitatively by a blinded investigator to describe general structural patterns rather than automated quantitative vessel density.

Immunostaining of tissue sections showed more abundant and better organized LYVE-1-positive lymphatic structures in the LEPC-treated group (Figure 4b). In contrast, MSC-treated and control tissues displayed fewer and less organized LYVE-1-positive structures within the excision region. These findings support a more favorable pattern of lymphatic tissue remodeling after LEPC treatment.

## 4. Discussion

In this study, the attrition observed in our cohort, particularly the 50% loss in the PBS control group, introduces a potential dropout bias. This selective loss may overrepresent animals with a milder baseline surgical trauma or stronger endogenous healing capacity, thus narrowing the observable therapeutic gap between groups and reducing statistical power for secondary endpoints.

We show that adipose-derived lymphatic endothelial progenitor cells (LEPCs) enhance both structural and functional recovery in a validated murine model of secondary lymphedema. Across independent readouts, LEPC-treated animals showed a consistent improvement in tail diameter, IVIS signal intensity, and qualitative LYVE-1 staining patterns compared with both PBS- and MSC-treated groups. The longitudinal mixed-effects analysis strengthened this interpretation by showing a significantly steeper increase in tail diameter over time in control animals relative to the LEPC group, whereas MSC-treated animals showed an intermediate trajectory without a significant difference versus LEPC. Overall, LEPC treatment produced concordant functional improvement (IVIS signal and tail diameter) together with more abundant and better organized LYVE-1-positive lymphatic structures, supporting a true regenerative effect in this model. The concordance between functional imaging, morphometric follow-up, and histological findings supports a true biological treatment effect rather than a measurement-specific artifact.

The sex-dependent pattern observed in the longitudinal tail-diameter analysis deserves careful consideration. In our model, the morphometric benefit of LEPC treatment was more evident in males, which showed an almost flat predicted temporal trajectory, whereas the reduction in tail swelling was less apparent in females. Although the present study was not designed to identify the mechanisms underlying sex-specific therapeutic responses, this finding is biologically plausible. Experimental evidence indicates that lymphatic function is modulated by sex hormone signaling, particularly through the estradiol–estrogen receptor α axis, which has been shown to protect against experimental lymphedema [34]. In addition, recent data suggest that iNOS-mediated nitric oxide production contributes to sex-based differences in secondary lymphedema severity, with male iNOS-deficient mice showing reduced swelling, inflammation, and tissue necrosis, whereas comparable protection was not observed in females [35]. These observations support the hypothesis that the inflammatory and nitrosative microenvironment after lymphatic injury may differ between sexes and could modulate edema progression and the regenerative performance of transplanted progenitor cells. Given the small size of these sex-specific strata, these findings are non-conclusive and must be interpreted strictly as exploratory and hypothesis-generating, and support the inclusion of sex as a biological variable in future preclinical studies of lymphatic regeneration. Because estrous cycle stage, circulating sex hormone levels, ERα signalling, macrophage polarization, and iNOS activity were not assessed in this study, no causal mechanism can be inferred from these data.

### 4.1. Methodological Considerations Supporting Translational Relevance

An important methodological aspect of this study is the use of IVIS-based near-infrared ICG imaging as the main functional readout. Conventional assessments, such as Evans blue transit, provide limited sensitivity and cannot be performed longitudinally. In contrast, IVIS enables repeated and quantitative assessment of tracer-associated fluorescence within the same animal, thereby improving reproducibility and translational value. Recent studies support the use of longitudinal near-infrared ICG imaging as a sensitive functional endpoint in preclinical lymphatic models [24]. In our dataset, IVIS-based quantification clearly distinguished LEPC-treated animals from MSC-treated and control groups, highlighting its utility for preclinical therapeutic screening.

The murine tail model employed in this study remains one of the most widely used and reproducible systems to evaluate lymphatic dysfunction and regeneration [21,22,23]. Its defined injury pattern, predictable edema kinetics, and compatibility with high-resolution imaging provide a stable platform for testing regenerative interventions. The integration of IVIS-ICG imaging into this model further enhances sensitivity and supports comprehensive evaluation of lymphatic recovery.

### 4.2. Regenerative Advantages of LEPCs over MSC- and ADSC-Based Therapies

Previous studies using MSCs or adipose-derived regenerative cell populations have demonstrated beneficial paracrine effects on lymphangiogenesis and tissue remodelling in experimental lymphedema [15,16]. Enhanced approaches such as Hippo–YAP modulation have further amplified the lymphangiogenic potential of adipose-derived stromal cells in murine tail lymphedema [18]. Our results expand this landscape by showing that LEPCs can induce substantial lymphatic regeneration without requiring gene engineering.

The podoplanin-positive progenitor population used here was associated with improved lymphatic repair, as shown by reduced edema and the presence of more abundant and better organized LYVE-1-positive lymphatic structures. These findings support the hypothesis that LEPC-mediated recovery may involve paracrine activity and/or direct endothelial incorporation, although the relative contribution of each mechanism cannot be determined in the absence of dedicated cell-tracking approaches. The pattern of structural remodelling observed is consistent with intussusceptive lymphangiogenesis, as recently described in high-resolution imaging studies [27].

### 4.3. Microenvironmental Barriers and Potential Combinatorial Strategies

Secondary lymphedema is characterized by a fibrotic and inflammatory milieu that can hinder tissue repair and limit long-term recovery [5,36,37,38]. In our study, LEPC-treated tissues showed a lower non-tissue/void area at day 36, consistent with reduced tissue disruption within the injury zone. In parallel, this finding may reflect less interstitial expansion and improved tissue organization in the treated group. Emerging evidence suggests that targeting extracellular matrix remodeling may potentiate regenerative strategies in secondary lymphedema. Inhibition of uPARAP has been reported to improve lymphatic architecture and reduce fibrosis in preclinical models [39]; mechanotransduction pathways such as Hippo–YAP/TAZ may also influence lymphatic endothelial behaviour [18]. In our study, the lower non-tissue/void area observed in LEPC-treated tissues supports the relevance of these microenvironmental constraints.

### 4.4. Paracrine Cooperation and Integration with Next-Generation Therapeutics

Reported synergy between MSCs and LEPCs suggests that paracrine support may enhance progenitor survival and lymphangiogenic activity; for example, MSC-derived IGF-1 has been linked to improved LEPC function via PI3K/Akt/mTOR signalling and superior outcomes with cotherapy compared with single-cell approaches [40]. Other “next-generation” combination strategies, such as Apelin + VEGF-C delivery via mRNA [41] and platelet-derived extracellular vesicles [42], further support a multimodal regenerative strategy. Together, these data raise the possibility that integrating LEPCs with paracrine-active or microenvironment-modulating interventions could improve durability beyond the functional benefit observed with LEPCs alone in our model, although this was not tested here.

### 4.5. Positioning of LEPC Therapy Within the Evolving Lymphatic Regeneration Landscape

LEPCs represent a mechanistically rational strategy for secondary lymphedema given their lymphatic-lineage orientation. In our study, LEPC treatment was associated with concordant functional improvement (IVIS signal and tail diameter) together with more abundant and better organized LYVE-1-positive lymphatic structures, supporting further translational evaluation. Key next steps include standardizing LEPC isolation and characterization workflows [43], testing whether combination approaches improve performance in fibrotic tissue, and extending follow-up to assess durability, biodistribution, and safety.

Beyond demonstrating local lymphatic regeneration in the tail, our findings should also be considered within the broader concept of tissue-specific lymphatic niches. Increasing evidence indicates that lymphatic vessels are shaped by the surrounding microenvironment and adopt organ-specific structural and functional properties in response to local stromal, vascular, and immune cues. Consequently, successful lymphatic regeneration is likely to depend not only on restoring vessel continuity but also on recapitulating the specialized niche that supports lymphatic identity and function. This concept is particularly relevant for complex macroenvironments such as the skeletal system, where lymphatic vessels interact with bone, periosteum, bone marrow, and specialized stromal populations to regulate tissue homeostasis, immune cell trafficking, and repair. Therefore, the regenerative response observed in the tail may represent a proof of principle that could be extended to more specialized organ-specific lymphatic niches, although additional studies will be required to determine whether LEPCs can integrate and function appropriately within these more complex tissue environments.

### 4.6. Limitations

Several limitations of this study must be acknowledged. First, the sample size, although consistent with established murine tail lymphedema studies [21,22,23], remains modest and may limit the statistical power for detecting small or late therapeutic effects. Increasing the group size, especially for MSCs, could help confirm the differences observed in this work.

Second, the tail model—despite being one of the most reproducible and widely used platforms for lymphatic research—does not fully recapitulate the anatomical and biomechanical complexity of human limb lymphedema. The absence of muscle compartments, fascia, and weight-bearing forces limits the extrapolation of certain mechanotransductive phenomena and stromal interactions.

Third, although IVIS-ICG provides a sensitive functional assessment, it reflects tracer-associated fluorescence within the ROI rather than direct measurement of lymphatic pumping or valve competence. Additional techniques, such as near-infrared dynamic contractility imaging or lymphoscintigraphy, could strengthen functional characterization.

Fourth, the identification of LEPCs relies on classical lymphatic lineage markers. While consistent with current standards, the heterogeneity of lymphatic progenitor populations is increasingly recognized [6,43]. A key limitation of our cell therapy protocol is the absence of long-term in vivo tracking of transplanted LEPCs. Consequently, we cannot determine whether the increased density of organized LYVE-1+ vessels stems from direct physical incorporation of the progenitors into the host capillaries or via potent transient paracrine actions that stimulate endogenous lymphangiogenesis prior to clearance. Future studies utilizing green fluorescent protein (GFP) line tracing are warranted to dissect these specific cellular dynamics. Additionally, a phenotypic characterization limit exists regarding our cell preparations: although isolation followed a validated lineage protocol, batch-specific post-sort flow cytometry profiles and purity metrics were not retained for the specific isolates injected in this study, which prevents direct quantification of batch-to-batch variability.

Fifth, LEPC–host interactions within the fibrotic microenvironment have not been investigated mechanistically. The contributions of macrophages, fibroblasts, and Th2-skewed inflammation to graft success or failure remain largely unexplored in this setting.

Finally, long-term durability and potential off-target effects could not be assessed because of the 36-day follow-up. Chronic models—beyond 8–12 weeks—are required to capture lymphatic remodelling, stabilize fibrosis, and maintain the persistence of engrafted cells.

### 4.7. Future Directions

Future work should prioritize mechanistic validation and translational strengthening. Key next steps include cell-tracking approaches to define the relative contribution of paracrine effects versus endothelial incorporation, extending follow-up to assess durability and safety, and testing clinically aligned models (e.g., hindlimb and/or irradiation-associated lymphedema). In parallel, standardization of LEPC isolation/characterization will be essential to improve reproducibility and support future translation [43].

## 5. Conclusions

Our findings indicate that LEPC transplantation improves multiple hallmarks of secondary lymphedema and is associated with more favorable functional, morphometric, and histological outcomes than MSC and PBS treatment in this preclinical model. Together, these results support further investigation of LEPC-based interventions as a regenerative strategy for secondary lymphedema, including studies designed to define mechanisms, durability, and translational feasibility.

## Figures and Tables

**Table 1:** Descriptive tail diameter values by treatment group, sex, and time point.

Time Point	Control Group Mean ± SD		MSC Group Mean ± SD		LEPC Group Mean ± SD	
	Female (*n* = 2)	Male (*n* = 2)	Female (*n* = 4)	Male (*n* = 3)	Female (*n* = 3)	Male (*n* = 3)
Baseline	3.14 ± 0.08	3.12 ± 0.13	3.13 ± 0.07	3.24 ± 0.08	3.08 ± 0.04	3.29 ± 0.04
Day 4	3.94 ± 0.32	4.75 ± 0.63	4.64 ± 0.88	4.53 ± 0.09	3.57 ± 0.45	4.44 ± 0.12
Day 7	4.40 ± 0.35	5.37 ± 0.15	4.92 ± 0.87	5.45 ± 0.17	3.96 ± 0.06	4.79 ± 0.27
Day 11	4.90 ± 0.69	5.99 ± 0.38	5.05 ± 0.66	5.31 ± 0.26	4.76 ± 0.11	5.07 ± 0.33
Day 14	5.07 ± 0.67	6.08 ± 0.72	5.07 ± 0.34	5.19 ± 0.34	5.01 ± 0.14	5.33 ± 0.26
Day 18	5.87 ± 0.39	6.33 ± 0.94	5.15 ± 0.37	5.56 ± 0.46	5.16 ± 0.21	5.35 ± 0.16
Day 21	6.09 ± 0.46	6.29 ± 0.56	5.04 ± 0.27	5.54 ± 0.87	5.44 ± 0.29	5.15 ± 0.19
Day 25	5.89 ± 0.23	6.26 ± 0.60	5.10 ± 0.59	5.58 ± 0.91	5.50 ± 0.35	5.16 ± 0.18
Day 28	5.34 ± 0.02	6.43 ± 0.04	5.13 ± 0.62	5.29 ± 0.77	5.47 ± 0.87	5.01 ± 0.58
Day 32	4.90 ± 0.21	6.43 ± 0.47	5.10 ± 0.55	5.35 ± 0.84	5.23 ± 0.69	4.56 ± 0.26
Day 36	4.72 ± 0.61	6.46 ± 0.51	4.90 ± 0.65	5.41 ± 1.33	4.81 ± 0.64	4.41 ± 0.28

Note. Data are presented as mean ± SD. Tail diameter is expressed in millimetres. Control Group refers to PBS-treated animals. Control Group: female *n* = 2, male *n* = 2; MSC Group: female *n* = 4, male *n* = 3; LEPC Group: female *n* = 3, male *n* = 3.

**Table 2:** Linear mixed-effects model for longitudinal tail diameter evolution.

Effect	Coefficient	*p* Value	95% CI
Fixed effects			
Group			
Control vs. LEPC	0.050	0.879	−0.595 to 0.695
MSC vs. LEPC	−0.007	0.982	−0.583 to 0.570
Sex			
Female vs. male	−0.733	0.013	−1.309 to −0.156
Group × Sex			
Control × Female	0.342	0.463	−0.570 to 1.254
MSC × Female	0.585	0.147	−0.205 to 1.375
Day	0.017	0.142	−0.006 to 0.040
Group × Day			
Control × Day	0.053	0.004	0.017 to 0.089
MSC × Day	0.020	0.217	−0.012 to 0.052
Sex × Day			
Female × Day	0.038	0.020	0.006 to 0.070
Group × Sex × Day			
Control × Female × Day	−0.064	0.014	−0.114 to −0.013
MSC × Female × Day	−0.046	0.039	−0.091 to −0.002
Constant	4.473	<0.001	4.065 to 4.881
Random effects	Estimate		95% CI
Var (Day)	0.00011		0.00003 to 0.00040
Var(residual)	0.40548		0.3278 to 0.5015

Note. Data are shown as model coefficients, *p* values, and 95% confidence intervals. The linear mixed-effects model included treatment group, sex, day, and their interaction terms as fixed effects, with a random slope for day at the individual-animal level. The reference category was LEPC-treated male mice. LEPC, lymphatic endothelial progenitor cell; MSC, mesenchymal stem cell; CI, confidence interval. The likelihood-ratio test comparing the mixed model with the linear model was significant: χ^2^ = 7.07, df = 2, *p* = 0.004.

## Data Availability

The datasets generated and/or analysed during the current study are available from the corresponding author on reasonable request.

## Abbreviations

ADSCs	Adipose-derived stem cells.
ARRIVE	Animal Research: Reporting of In Vivo Experiments.
ECGM	Endothelial cell growth medium.
H&E	Hematoxylin and eosin.
ICG	Indocyanine green.
IL	Interleukin.
IVIS	In vivo imaging system.
LEPCs	Lymphatic endothelial progenitor cells.
LVA	Lymphaticovenular anastomosis.
LYVE-1	Lymphatic vessel endothelial hyaluronan receptor 1.
MSCs	Mesenchymal stem cells
PBS	Phosphate-buffered saline.
PROX1	Prospero homeobox 1.
ROI	Region of interest.
SPF	Specific pathogen-free.
UPV/EHU	University of the Basque Country.
VLNT	Vascularized lymph node transfer.
